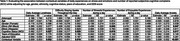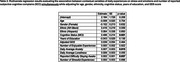# How Daily Experiences of Stress and Emotions Contextualize Subjective Cognitive Concerns: Findings from Daily Digital Diary Assessments in the Einstein Aging Study (EAS)

**DOI:** 10.1002/alz70857_107552

**Published:** 2025-12-25

**Authors:** Angel Garcia De La Garza, Carol A. Derby, Cuiling Wang, Nelson A. Roque, Mindy J. Katz, Richard B. Lipton, Laura A. Rabin

**Affiliations:** ^1^ Albert Einstein College of Medicine, Bronx, NY, USA; ^2^ The Pennsylvania State University, University Park, PA, USA; ^3^ Brooklyn College of the City University of New York, Brooklyn, NY, USA; ^4^ The Graduate Center, CUNY, New York, NY, USA

## Abstract

**Background:**

Ecological momentary assessment (EMA) is increasingly used to track subjective cognitive concerns (SCC), or self‐perceived memory and cognitive difficulties, in real time. This study examines how daily contextual factors—such as anxiety, stress, socialization, enjoyment, and wakefulness—influence SCC in EMA diaries. While SCCs predict future cognitive decline, their moment‐to‐moment relationship with daily experiences remains unclear. Using smartphone‐based EMA, we explore associations between daily experiences and SCC in community‐dwelling older adults. EMA captures within‐person fluctuations, minimizing recall bias and facilitating assessment of transient factors that may influence SCCs.

**Methods:**

Participants from the EAS completed six surveys daily over 14 days. Cognitive lapses were assessed once per day in the evening survey, where participants also rated their difficulty staying awake (0–100). Across six surveys throughout each day, they reported if they experienced enjoyable or stressful activities since the last survey. Participants also reported their current levels of anxiety and loneliness at the time of the survey. We calculated daily counts of stressful and enjoyable events and daily averages of anxiety and loneliness. Using linear mixed‐effects models, we examined the day‐level cross‐sectional association of contextual variables with SCCs, adjusting for age, sex, race/ethnicity, depression status, and cognitive status (cognitively unimpaired or mild cognitive decline). We also conducted a multivariable analysis incorporating these contextual variables into one model.

**Results:**

Analyses included 310 community‐dwelling participants (mean age = 77.5, SD = 4.94; 66.4% female; 47.1% non‐Hispanic White, 40.7% non‐Hispanic Black, 12.3% Hispanic; 70% cognitively unimpaired), contributing 11.40 diary days on average. Difficulty staying awake (*p* = 0.0159), average stress (*p* < 0.001), loneliness (*p* < 0.001), and anxiety (*p* < 0.001) were positively associated with SCCs. When examining these contextual variables simultaneously, difficulty staying awake (*p* = 0.037) and anxiety (*p* = 0.015) remained independently associated with daily total reported SCCs.

**Conclusions:**

Contextual variables of daily experiences on stress and emotions are associated with SCCs. Future work should incorporate these variables when using SCC as a screening tool for cognitive impairment risk. We plan to study individual vulnerabilities to contextual variables as predictors of longitudinal cognitive decline.